# Clinical Prediction of Type 2 Diabetes Mellitus (T2DM) via Anthropometric and Biochemical Variations in *Prakriti*

**DOI:** 10.3390/diseases10010015

**Published:** 2022-03-03

**Authors:** Shriti Singh, Neeraj Kumar Agrawal, Girish Singh, Sangeeta Gehlot, Santosh Kumar Singh, Rajesh Singh

**Affiliations:** 1 Department of Kriya Sharir, Faculty of Ayurveda, Institute of Medical Sciences, Banaras Hindu University, Varanasi 221005, India; sangeetagehlot@gmail.com; 2 Department of Endocrinology and Metabolism, Institute of Medical Sciences, Banaras Hindu University, Varanasi 221005, India; drnkavns@gmail.com; 3 Centre of Biostatistics, Institute of Medical Sciences, Banaras Hindu University, Varanasi 221005, India; drgirishsingh@yahoo.com; 4 Department of Microbiology, Biochemistry, and Immunology, Cancer Health Equity Institute, Morehouse School of Medicine, 720 Westview Drive SW, Atlanta, GA 30310, USA; sksingh@msm.edu

**Keywords:** *Vata*, *Pitta*, *Kapha*, biochemical marker, T2DM, anthropometric parameters

## Abstract

Type 2 Diabetes Mellitus (T2DM) is a complicated multifactorial illness involving hereditary and external environmental variables. The symptoms typically appear gradually over a number of years without realizing it. This viewpoint is further supported by the Ayurvedic constitution concept (*Prakriti*). *Prakriti* explains the biological variability that is observed in different individuals. This study was conducted a retrospective investigation to examine if there was a link between type 2 diabetes and an individual’s constitution based on anthropometric and biochemical characteristics. Physical and mental characteristics and anthropometric and biochemical markers were used to determine reported cases’ prevailing *Dosha Prakriti* (constitution). Based on biochemical and anthropometric data, significant differences in *Prakriti* were found between the case (T2DM patients) and control (person without diabetes) groups. The incidence of numerous secondary problems linked with T2DM patients was also evaluated according to their *Prakriti* types, which revealed a positive relationship. The three primary contributing parameters, such as waist-hip ratio, postprandial blood sugar, and serum creatinine, were correctly classified all person with or without diabetes subjects to 90.6% of the time, whereas the constitution-wise study classified person with diabetes and without diabetes individuals of *Pitta* and *Kapha Prakriti* to 94.3% and 90%, respectively. A discriminant function was created to predict a person with diabetes and without diabetes based on these three contributing factors. The primary contributing biochemical parameters discovered by *Prakriti* in the current study could be used as a biochemical disease diagnostic for predicting type 2 diabetes susceptibility.

## 1. Introduction

Diabetes mellitus (DM) is wreaking havoc on the public health system. DM is linked to an elevated risk of numerous types of diseases and malignancies [1]. The disease’s prevalence is anticipated to rise to 623 million by 2045, with type 2 diabetes mellitus (T2DM) accounting for about 90% of cases [2]. Hyperglycemia is a symptom of T2DM caused by insulin resistance and a decrease in insulin production. It is a common disorder with increasing prevalence due to lack of physical activity and increased degrees of obesity [3]. Insulin resistance is a condition that affects body cells such as the muscle, liver, and fat cells; consequently, they fail to respond to insulin, even when insulin levels are high. In fat cells, triglycerides are broken down to produce free fatty acids for energy; muscle cells are deprived of energy sources, and liver cells fail to build up glycogen stores. Several factors impede understanding of the pathophysiology of type 2 diabetes [4]. In diabetes mellitus, hyperglycemia initiates and sustains an injury to many organs and systems, resulting in serious complications such as retinopathy, neuropathy, cardiovascular diseases, nephropathy, peripheral vascular diseases, and periodontal pathologies [5].

Ayurveda is an ancient Indian healing system that uses a personalized approach that has been documented and practiced for thousands of years. Ayurveda has a unique classification of the human population based on the individual constitution or *Prakriti* [6]. The *Tridosha* theory of Ayurveda’s recognizes principles of movement (*Vata*), metabolism (*Pitta*), and strength (*Kapha*) as distinct phenotypic groupings. As per this system, every individual is born with their basic constitution, which determines to a great extent inter-individual variability in susceptibility to diseases and response to the external environment, diet, and drugs. According to Ayurveda, there are five elements (panchmahabhoot) Aakash (space), Jala (water), Agni (fire), Prithvi (earth), and Vayu (air); its combination is responsible for the formation of dosha, i.e., *Vata* (air and space), *Pitta* (fire and water), *Kapha* (water and earth). Because dosha balance is a sign of good health and imbalance is a sign of disease, each person’s dosha proportion is different. According to Ayurveda, an individual’s *Prakriti*, or constitution, is determined by the relative predominance of *Vata, Pitta,* and *Kapha*. *Prakriti* refers to the sum of all physical, physiological, and psychological traits. People with different *Prakriti* types react differently to the same stimuli. *Prakriti* has been extensively studied to understand better its genomic and biochemical correlations [7,8,9]. The prior knowledge of *Prakriti* of individuals/patients helps suggest lifestyle modifications in patients. According to Ayurveda, the manifestation of diabetes occurs due to *Kapha’s* predominant diet, such as sweets, curd, newly harvested grains, etc. As a result, the knowledge of patients’ dominant *Dosha Prakriti* aids in diagnosis, prognosis, and treatment of their disease. Ayurveda describes DM under non-insulin-dependent DM as *Kaphaj* and *Pittaj Prameha;* it represents early diabetes and acute diabetes. Based on complications associated with the chronic stage of the disease, the same T2DM condition is termed *Vataja Prameha*/*Madhumeha*. Similarly, insulin-dependent DM has also been defined under *Vataja Prameha* [10]. *Prakriti* types exhibit striking differences in biochemical and hematological parameters [11].

The present study focused on determining the prevalence of dominant *Prakriti* in T2DM patients in relation to their anthropometric and biochemical variables.

## 2. Material and Methods

### 2.1. Sample Size

The sample size was determined by estimating some key variables from previous research [12,13]. The sample size was calculated using relevant anthropometric and biochemical parameters, with alpha set at 5% and power set at 90%. A total of 112 subjects for the case and 112 subjects for the control group were the final sample size for the study.

### 2.2. Design

This study included 112 persons with diabetes who visited the outpatient section of the Endocrine and Metabolism department existing in Sir Sunderlal Hospital, Institute of Medical Sciences, Banaras Hindu University (B.H.U.), Varanasi, India, and 112 healthy (control subjects) volunteers were drawn from B.H.U employee’s community from different departments. The research was carried out with random selection between February 2016 and April 2017. All the subjects were registered after obtaining their written consent. *Prakriti* assessment was carried out by a questionnaire developed by Tripathi et al. 2019 [14]. The short description of the *Prakriti* assessment tool is shown in Supplementary Table S1. The characteristics of *Vata, Pitta*, and *Kapha* dominant *Prakritis* were studied according to Ayurvedic scriptures. Research work has been approved by the Institutional Ethics Committee vide letter No. Dean/2015–2016/1572 dated 30 December 2015.

### 2.3. Criteria of Subject Selection

Subjects of both sexes over the age of 40 years were registered. Informed written consent was taken from them. 

#### 2.3.1. Selection of Diabetic Subjects Was Based on the Following Criteria

(i) Fasting blood glucose value 126 mg/dL or higher.
(ii) Post Prandial blood glucose level more than 200 mg/dL.
(iii) No insulin-dependent cases (type 1 diabetes).

Fasting blood glucose value was measured two times. Additionally, 75 g OGTT (Oral Glucose Tolerance Test) was conducted. Additionally, the blood samples were collected after 2 h of the meal. All tests were conducted in the Center for Clinical Investigation (CCI) Laboratory, Banaras Hindu University, India.

#### 2.3.2. Selection of Control Subjects

(i) No family history of type 2 diabetes (because of this, there are genetically fewer chances to have predisposition with the disease).
(ii) Blood glucose level within the normal range, i.e., Fasting and Postprandial. (It shows glucose metabolism and insulin action were proper; hence no hyperglycemia was detected).
(iii) Healthy individuals, not under any medication, were considered. (Biochemical parameters may be affected due to intake of medication).

#### 2.3.3. Exclusion Criteria

(i) Unwilling participants.
(ii) Patients with insulin-dependent DM, tuberculosis, AIDS, and malignancies were excluded.

Cases of diagnosed T2DM in both sexes ranging in age from 40 to 70 years were chosen randomly from February 2016–April 2017. For the *Prakriti* assessment of T2DM patients, pre diseased conditions of their health and habits were considered. Clinically type 2 diabetes persons have been considered a case for the study, whereas clinically, persons without diabetes acted as a control. For this study, the standard criteria of the American diabetic association 2015 for diabetes were adopted. A 3 mL blood sample from the antecubital vein was collected from each case and control subjects in an EDTA vial. 

#### 2.3.4. Limitations

The study covers only a small population of eastern Uttar Pradesh, India. The laboratory made all tests and patients came up with a report. In the future, to obtain more precise results, the study may be conducted with a large sample size, and samples may be collected from a different area of the country or worldwide.

### 2.4. Parameters Associated with Study

Various anthropometric, biochemical, and physiological parameters were adopted for case and control subjects under study. Height, weight, BMI, and waist-hip ratio were taken as anthropometric parameters while blood pressure was used as a physiological criterion; in contrast, blood sugar fasting, postprandial fasting lipid profile, and serum creatinine were assessed under biochemical parameters. eGFR was calculated by online software through the Modification of Diet in Renal Disease (MDRD) study equation for estimating the risk of kidney damage. For T2DM individuals, HbA1c and ECG were also recorded. Further investigating any secondary complication in T2DM cases, retinopathy, neuropathy, family history, and macro proteinuria were evaluated.

### 2.5. Statistics

*Prakriti*-based statistical analysis of clinical data was performed. Data were represented as Mean± SD. Because just one subject of *Vata Prakriti* was recorded in T2DM cases, it was excluded from the statistical analysis, and only *Pitta* and *Kapha Prakriti* samples were used. In the present study, for T2DM cases, the independent group *t*-test was applied. For the control group, one-way ANOVA was used, and to observe the significance among different *Prakriti* groups; the Post hoc test was applied. Bonferroni test was applied following ANOVA. For secondary complications in the case group, i.e., Neuropathy, Retinopathy and family history of CVD, etc., their association with *Prakriti* was determined by the chi-square test, which shows the significance of incidence among different *Prakriti*. *p*-values < 0.05 were considered for statistical significance in all the statistical analyses. Discriminant function analysis was applied to see major contributing parameters in *Pitta* and *Kapha Prakriti* related to disease in the case and control group.

## 3. Results

Among a total of 112 T2DM cases, *Pitta* (n = 51) and *Kapha* (n = 60) individuals were grouped according to the dominance of their *Dosha Prakriti.* Patients with *Kapha Prakriti* were found to be more than those with *Pitta Prakriti* and *Vata Prakriti* (n = 1). Similarly, 112 persons without diabetes were grouped into *Vata* (n = 17), *Pitta* (n = 55), and *Kapha* (n = 40) based on their dominance of *Dosha Prakriti*. In individuals with diabetes, the average value of age was found 52.9 years, and for control individuals, it was 50.2 years. Gender proportion for persons with diabetes females (n = 41) 37% and diabetes males (n = 70) 63% and in persons without diabetes females (n = 26) 23% and males (n = 86) 77%. The average duration of suffering from diabetes was 5.8 years in patients.

Among finding associated with *Prakriti*-based anthropometric and biochemical parameters for type2 diabetes patients, they showed statistically significant differences in parameters, i.e., weight, Basal Metabolic Index (BMI), waist-hip ratio, fasting, and postprandial blood sugar, systolic blood pressure, total cholesterol, HDL and LDL, between *Kapha* and *Pitta Prakriti*. Despite the fact that the mean values of height, serum triglyceride, serum creatinine levels, and HbA1c were greater in *Kapha Prakriti*, diastolic blood pressure, and eGFR values were higher in *Pitta Prakriti*; there was no statistically significant difference found between the two *Prakriti* (Table 1). 

A statistically significant difference was found in BMI, systolic and diastolic blood pressure, fasting and postprandial blood sugar, LDL, triglyceride, total cholesterol, serum creatinine, and eGFR parameters when compared anthropometric and biochemical parameters in individuals with diabetes cases and without diabetes control group individuals belonging to the *Kapha*-dominant *Prakriti.* Similarly, in *Pitta*-dominant *Prakriti* individuals with diabetes, and in non-diabetics, their weight, systolic and diastolic blood pressure, fasting and postprandial blood sugar, serum creatinine, and eGFR parameters showed statistically significant differences (data shown in Supplementary Tables S2 and S3).

No significant difference was found in secondary complications associated with T2DM between the *Prakriti* groups. Based on mean values, this study observed that more patients have cardiovascular problems than those belonging to *Kapha Prakriti* than *Pitta Prakriti*. The incidence of retinopathy, neuropathy and micro and macro proteinuria differed significantly between *Pitta* and *Kapha Prakrit patients*. In contrast, the incidence of family history of cardiovascular disease and family history of diabetes showed a higher correlation in patients of *Kapha Prakriti*, i.e., 25% and 53%, respectively, whereas in patients with *Pitta* *Prakriti*, these incidences were 45.1% and 19.6%, respectively. At the same time, no significant differences were observed among them (Table 2).

In the group without diabetes (control group) consisting of all three dominant *Prakriti* types, all the parameters showed a significant difference among the three dominant *Prakriti* groups except serum triglyceride, total cholesterol, and GFR values. On applying Post hoc test; height, weight, BMI, and waist-hip ratio parameters showed significant differences among a comparison of *Vata* vs. *Pitta* and *Vata* vs. *Kapha,* respectively (*p* < 0.05), whereas in *Pitta* vs. *Kapha* comparison, *Prakriti* individuals did not show a significant difference for parameters as mentioned below (Table 3). Systolic and diastolic blood pressure showed significant variations in all the combinations of the *Prakriti* group. In fasting blood sugar, a significant difference was noted in all pair-wise comparisons of *Prakriti,* i.e., *Vata* vs. *Pitta, Pitta* vs. *Kapha, Vata* vs. *Kapha,* whereas in postprandial blood sugar, the comparison of *Vata* vs. *Pitta Prakriti* did not show a significant difference. There was no significant difference between HDL levels in the *Vata* vs. *Pitta* combination in the fasting lipid profile.

On the other hand, *Vata* vs. *Kapha* and *Pitta* vs. *Kaph* showed a substantial difference. A significant difference was found in LDL among *Vata* vs. *Kapha* and *Pitta* vs. *Kapha Prakriti* individuals (*p* < 0.05). No significant difference was found in total cholesterol, triglyceride, and eGFR in any combination of *Prakriti.* In contrast, a significant difference was noted in serum creatinine values for *Vata* vs. *Pitta* and *Vata* vs. *Kapha Prakriti* groups (Table 3).

Discriminant function analysis was carried out on the case (persons with diabetes) vs. control group (without diabetes), based on some important biochemical parameters. Among all the parameters considered in the present study, the three parameters were major contributing, e.g., waist-hip ratio, postprandial blood sugar, serum creatinine, which resulted in a 90.6% correct classification of all cases and control subjects. The standardized canonical discriminant function coefficient of waist-hip ratio (WHR), postprandial blood sugar (PPBS), and serum creatinine (Scr) for total subjects were 0.290, 0.973, and 0.622, respectively. The classification function coefficients are as follows:

F_Totalcase_ = −87.669 + 161.368 WHR + 0.045 PPBS + 13.16 Scr

F_Totalcontrol_ = −84.355 + 171.303 WHR+ 0.010 PPBS + 6.255 Scr

The subjects were classified, i.e., individuals with diabetes (cases) or without diabetes (control), based on higher values among the above two functions. The discriminant function analysis was carried out on different *Prakriti* individuals (*Pitta and Kapha*) (data shown in Supplementary Tables S2 and S3). These three above-mentioned significant contributors showed correct classification for *Pitta* and *Kapha Prakriti* individuals in the present study by the value of 94.3% and 90.0%, respectively. The standardized canonical discriminant function coefficient of waist-hip ratio (WHR), postprandial blood sugar (PPBS), and serum creatinine (Scr) for *Pitta Prakriti* individuals were 0.530, 0.908, 0.483, respectively, whereas for *Kapha Prakriti*, it was 0.100, 1.031, 0.678, respectively.

The classification function coefficient for *Pitta Prakriti* is as follows:

F_Pitta case_ = −102.429 + 197.221 WHR + 0.085 PPBS + 11.062 Scr

F_Pitta control_ = −108.854 + 221.572 WHR + 0.024 PPBS + 4.457 Scr

The classification function coefficient for *Kapha Prakriti* is as follows:

F_Kapha case_ = −122.037 + 208.069 WHR + 0.81 PPBS + 19.07 Scr

F_Kapha control_ = −106.443 + 204.455 WHR + 0.051 PPBS + 12.323 Scr

By comparing function coefficient for *Kapha* and *Pitta Prakriti,* we found that as compared with postprandial blood sugar and serum creatinine, the contribution of the waist-hip ratio was lower for *Kapha Prakriti* individuals as compared with *Pitta Prakriti*, it shows that the difference between the values of the waist-hip ratio of the *Kapha Prakriti* case (persons with diabetes) and control (group without diabetes) is smaller than that of *Pitta Prakriti* case and control. Similarly, serum creatinine contributes less to *Pitta Prakriti* than *Kapha Prakriti*, showing less difference between case and control values for this parameter in *Pitta Prakriti*.

## 4. Discussion

The *Prakriti* of patients determines the majority of diseases and their clinical manifestations. According to Ayurveda, *Vata, Pitta,* and *Kapha* (*Tridosha*) regulate all the physiological processes in the body. It also directly relates to an individual’s ability to fight sickness. The equilibrium of *Tridoshas* leads to perfect health, and disharmony leads to the diseased condition. In this study, the *Vata Prakriti* cases for type 2 diabetes were not encountered much. Only one case was found; this might be because only chronic cases of T2DM become converted into *Vatic Prameha* or type1 diabetes case where patients are completely insulin dependent as reported by Sharma and Chandola 2011 [10]. Individuals with diabetes and without diabetes in the present study were of similar age groups, which was over 40 years of age; the American Diabetes Association (ADA) recommends annual diabetes screening tests after people reach the age of above forty. The T2DM participants were *Pitta* and *Kapha Prakriti*. Height, weight, BMI, and waist-hip ratio were higher in *Kapha Prakriti* individuals than *Pitta* (Table 1). According to Charak, *Kapha Prakriti* individuals have a well-built and strong body than other *Prakriti* individuals [15]. The waist-hip ratio and BMI are both associated with insulin resistance [16], which is linked to T2DM. In *Kapha Prakriti’s* individuals, BMI and waist-hip ratio were higher above the physiological limit. In the *Kapha Prakriti* subjects, fasting and postprandial blood sugar were also higher, with a range of 182.90 ± 77.70 and 289.63 ± 112.13, respectively. T2DM is mainly *Kapha* dominant metabolic disorder in which digestive power becomes imbalanced, leading to toxin formation (Aama) in the body and ultimately causing improper glycemic control [12].

In people with diabetes, blood pressure is lower in *Kapha Prakriti* than in *Pitta Prakriti*, whereas lipid profile levels are more noteworthy in *Kapha Prakriti* than in *Pitta Prakriti*. In research by Amin et al. (2015), persons with *Kapha*-dominant *Prakriti* had a more extensive range (within normal limits) of cholesterol, triglycerides, VLDL, HDL, and LDL [17]. Serum creatinine is found higher in *Kapha Prakriti* individuals than *Pitta Prakriti*; this is because in *Kapha Prakriti*, aama formation due to impaired digestion and metabolism may lead to a higher level of serum creatinine [12]. In the present study, HbA1c was also found slightly higher in *Kapha Prakriti* persons but did not show a significant difference with *Pitta Prakriti* individuals. Additionally, Ayurveda addresses that Rakta (blood) and *Pitta* complement each other [18]. These studies suggest a higher hemoglobin level in *Pitta* than *Kapha* and *Vata Prakriti*.

The *Prakriti* of individuals is fixed, just like our genetic setup. This influences an individual’s physiological, physical, and psychological aspects, either in a healthy condition or in a diseased state. This is what the current study attempted to investigate. We found T2DM patients with *Pitta* and *Kapha Prakriti* exhibited differences in the parameters used for analysis in the same disease condition, confirming the above premise of *Prakriti-*based variances in distinct *Prakriti* types. 

Secondary complications associated with type 2 diabetes patients (Table 2) revealed a higher incidence of cardiovascular disorder (CVD), a family history of diabetes, and a family history of CVD in *Kapha Prakriti* individuals than in *Pitta Prakriti.* In contrast, the incidence of neuropathy, retinopathy, and micro and macro proteinuria was similar in *Pitta* and *Kapha Prakriti.*

Furthermore, Mahalle et al. (2012) have reported a strong relationship between cardiovascular disease or risk factors with *Vata Kaph* and *Kapha Prakriti* [19]. The presence of other microvascular complications, such as nephropathy or retinopathy acting as a risk factor for people with diabetic peripheral neuropathy, shows that these complications go hand-in-hand in those patients [20].

In the group without diabetes (control subjects), all the three *Vata, Pitta*, and *Kapha* dominant *Prakriti* individuals were detected. The height, weight, BMI, and waist-hip ratio were higher in *Kapha* than *Pitta* and lowest in *Vata Prakriti*. According to Ayurveda, *Vata Prakriti* individuals are lean, thin, and short stature. In contrast, *Kapha Prakriti* individuals have well-shaped, well-developed, and compact bodies, which may be responsible for the lower height and weight of the *Vata* individuals than *Kapha Prakriti* individuals [15]. Singh et al. (2013) noted maximum BMI in *Kapha Prakriti* individuals while minimum in *Vata Prakriti* individuals [21]. This finding confirms the present study; our results were also supported by the study of Tiwari and Gehlot (2015) [22] and Patel et al. (2012) [13].

Furthermore, systolic and diastolic blood pressure was highest in *Vata* than *Pitta* and least in *Kapha Prakriti* individuals; however, it is within the physiological limits. *Vata* dominant *Prakriti* patients had a relatively higher physiological limit of blood pressure. As per the concepts of Ayurveda, vitiated *Vyana Vata* (a type of body *Dosha*, running all over the body, responsible for blood circulation) may be responsible for the rise in blood pressure [23]. The values of blood sugar levels were also found higher in *Kapha* than *Pitta* and *Vata Prakriti* but within the physiological limit. Individuals with a *Kapha Prakriti* had a higher lipid profile than those with a *Pitta Prakriti*, whereas those with a *Vata Prakriti* had the lowest. These findings are supported by research by Kahatri and Sharma (2013) [24]. Moreover, serum creatinine level is higher in *Kapha Prakriti* individuals than *Pitta* and *Vata Prakriti* subjects. eGFR also showed a similar relation as serum creatinine, supported by a study by Amin and Sharma (2015) [17].

Anthropometric and biochemical indicators were shown to correlate significantly with *Prakriti* types in patients with diabetes and without diabetes. Individuals’ age and gender impact biochemical parameters, but we focused on *Prakriti*-based alterations in this study; even after accounting for the general impact of age and gender, *Prakriti*-wise substantial differences were discovered. Most diabetes-related metrics have a strong connection in *Kapha Prakriti*, indicating that *Kapha Prakriti* individuals are more prone to disease than other *Prakriti* types. This association is more precise when using discriminant function analysis on a few key contributing characteristics, resulting in a case and control group classification accuracy of over 90%. The current study produced a mathematical model that may be utilized to predict individuals with or without diabetes based on their waist-hip ratio, postprandial blood sugar, and serum creatinine (the three key contributing parameters of the present study) as their *Prakriti* types. In the current investigation, these three primary contributing characteristics were strongly linked to the onset of diabetes. In addition, central obesity is directly linked with a higher waist-hip ratio, higher postprandial blood sugar, and serum creatinine, all of which are associated with improper metabolism, which is the leading cause of T2DM.

## 5. Conclusions

This study found a positive association between various biochemical and anthropometric parameters related to *Prakriti* types of individuals in both case and control groups. Individuals *Prakriti* and a few key contributing characteristics associated with the disease could be exploited as *Prakriti-*based biochemical markers. In addition, a mathematical model was developed based on *Prakriti* and a few significant contributing characteristics to predict individuals with and without diabetes, which is a preliminary study in this approach. Prior awareness of *Prakriti* prone to particular diseases can aid in disease prevention in the early stages. The finding can be used to modify patients’ diet, lifestyles, and various medications to help them recover swiftly from illness. The decoding of the Ayurvedic concept of *Prakriti* could lead to global acceptance of Ayurvedic vocabulary.

In prospect of the present study, we believe that incorporating ancient Ayurvedic knowledge of *Prakriti* and the related concept of disease manifestation could benefit practitioners and the current healthcare system. Understanding *Prakriti-*based biochemical markers may open new avenues for scientific research into disease prognosis and treatment selection.

## Figures and Tables

**Table 1:** Showing anthropometric and biochemical parameters of type 2 diabetes subjects as per *Prakriti.*

Parameters	*Pitta* (n = 51) (Mean ± SD)	*Kapha* (n = 60) (Mean ± SD)	*t*-Test	*p*-Value
Height (cm)	158 ± 7	160 ± 10	1.1	0.26
Weight (kg)	64 ± 11	71 ± 10	3.5	0.001
BMI (kg/m^2^)	26 ± 4	28 ± 4	2.8	0.006
Systolic Blood Pressure (mm of Hg)	135 ± 16	124 ± 6	5.1	<0.001
Diastolic Blood Pressure (mm of Hg)	82 ± 10	79 ± 8.7	1.5	0.14
Waist Hip Ratio (cm)	0.9 ± 0.05	0.96 ± 0.05	8.2	<0.001
Fasting Blood Sugar (mg/dL)	142 ± 31	183 ± 78	3.5	0.001
Post Prandial Blood Sugar (mg/dL)	220 ± 64	290 ± 112	3.9	<0.001
HDL (mg/dL)	39 ± 7	44 ± 8	3.1	0.002
LDL (mg/dL)	107 ± 43	126 ± 38	2.5	0.01
Triglyceride (mg/dL)	137 ± 60	164 ± 69	1.9	0.05
Total Cholesterol (mg/dL)	167 ± 45	189 ± 40	2.7	0.007
SerumCreatinine (mg/dL)	0.95 ± 0.30	0.99 ± 0.31	0.7	0.48
eGFR * (mL/min/1.73 m^2^)	83 ± 40	77 ± 26	0.9	0.38
HbA1C (%)	8 ± 1.6	8 ± 2	1.0	0.31

* GFR—Glomerular Filtration Rate.

**Table 2:** Showing secondary complications and family history of the person with diabetes as per *Prakriti.*

Parameters	*Pitta Prakriti* (n = 51)	*Kapha Prakriti* (n = 60)	Between *Prakriti* Comparison Chi Square and *p* Value
Diabetes Retinopathy			
BDR *	11(21.6%)	13 (21.7%)	χ^2^ = 1.17
NPDR **	9(9.8%)	10 (6.6%)	*p* = 0.558
WNL ***	35 (68.6%)	37 (61.7%)	
Diabetes Neuropathy			
NO	15 (29.4%)	17 (28.3%)	χ^2^ = 0.0156
YES	36 (70.6%)	43 (71.70%)	*p* = 0.901
Cardiovascular disease			
NO	45(88.2%)	51(85.0%)	χ^2^ = 0.247
YES	6 (11.8%)	9 (15.0%)	*p* = 0.619
Micro, Macro proteinuria			
Macro uria	6 (11.8%)	4 (6.7%)	χ^2^ = 1.52
Micro uria	1 (2.0%)	3 (5.0%)	*p* = 0.469
Nil	44 (86.3%)	53 (88.3%)	
Family history of diabetes			
NO	28 (54.9%)	28 (46.7%)	χ^2^ = 0.748
YES	23 (45.1%)	32(53.3%)	*p* = 0.387
Family history of cardiovascular disorder			
NO	41 (80.4%)	45 (75.0%)	χ^2^ = 0.459
YES	10 (19.6%)	15 (25.0%)	*p* = 0.498

* BDR—Background diabetes retinopathy. ** NPDR—Non-proliferative persons with diabetes retinopathy. *** WNL—Within the normal limit.

**Table 3:** Showing anthropometric and biochemical parameters of the group without diabetes subjects as per *Prakriti*.

Parameters	*Vata Prakriti* (n = 17) Mean ± SD	*Pitta Prakriti* (n = 55) Mean ± SD	*Kapha Prakriti* (n = 40) Mean ± SD	F	*p*-Value	Post hoc Test (<0.05)
Height (cm)	157 ± 8	163 ± 8	165 ± 6	8.752	<0.0001	*Vata* vs. *Pitta* 0.004 *Vata* vs. *Kapha* < 0.001 *Pitta* vs. *Kapha* 0.480
Weight (kg)	55 ± 10	63 ± 10	68 ± 10	140.00	<0.0001	*Vata* vs. *Pitta* < 0.001 *Vata* vs. *Kapha* < 0.001 *Pitta* vs. *Kapha* 1.000
BMI (kg/m^2^)	22 ± 3	24 ± 3	25 ± 3	9.779	<0.0001	*Vata* vs. *Pitta* < 0.001 *Vata* vs. *Kapha* 0.009 *Pitta* vs. *Kapha* 0.300
Systolic Blood Pressure (mm of Hg)	126 ± 4	118 ± 4	115 ± 4	56.003	<0.0001	*Vata* vs. *Pitta* < 0.001 *Vata* vs. *Kapha* < 0.001 *Pitta* vs. *Kapha* 0.001
Diastolic Blood Pressure (mm of Hg)	87 ± 3	79 ± 4	76 ± 4	46.361	<0.0001	*Vata* vs. *Pitta* < 0.001 *Vata* vs. *Kapha* < 0.001 *Pitta* vs. *Kapha* 0.019
Waist Hip Ratio (cm)	0.9 ± 0.06	0.9 ± 0.08	0.95 ± 0.09	4.943	0.009	*Vata* vs. *Pitta* 0.018 *Vata* vs. *Kapha* 0.009 *Pitta* vs. *Kapha* 1.000
Blood Sugar (mg/dL) Fasting	83 ± 5	91 ± 8	97 ± 8	22.762	<0.0001	*Vata* vs. *Pitta* 0.001 *Vata* vs. *Kapha* < 0.001 *Pitta* vs. *Kapha* < 0.001
Blood Sugar (mg/dL) Post Prandial	109 ± 6	116 ± 10	123 ± 15	9.758	<0.0001	*Vata* vs. *Pitta* 0.072 *Vata* vs. *Kapha* < 0.001 *Pitta* vs. *Kapha* 0.015
HDL (mg/dL)	39 ± 3	41 ± 4	44 ± 4	10.683	<0.0001	*Vata* vs. *Pitta* 0.169 *Vata* vs. *Kapha* < 0.001 *Pitta* vs. *Kapha* 0.003
LDL (mg/dL)	94 ± 22	97.78 ± 18.50	99 ± 20	0.354	0.703	*Vata* vs. *Pitta* 0.169 *Vata* vs. *Kapha* <0.001 *Pitta* vs. *Kapha* 0.003
Triglyceride (mg/dL)	116 ± 9	122 ± 12	125 ± 14	2.573	0.081	*Vata* vs. *Pitta* 1.000 *Vata* vs. *Kapha* 1.000 *Pitta* vs. *Kapha* 1.000
Total cholesterol (mg/dL)	149 ± 24	161 ± 17	165 ± 18	3.861	0.024	*Vata* vs. *Pitta* 0.290 *Vata* vs. *Kapha* 0.076 *Pitta* vs. *Kapha* 1.000
Serum creatinine (mg/dL)	0.73 ± 0.08	0.79 ± 0.08	0.80 ± 0.07	6.224	0.003	*Vata* vs. *Pitta* 0.007 *Vata* vs. *Kapha* 0.003 *Pitta* vs. *Kapha* 1.000
eGFR * (mL/min/1.73 m^2^)	98 ± 8	99 ± 7	100 ± 8	0.648	0.525	*Vata* vs. *Pitta* 1.000 *Vata* vs. *Kapha* 0.825 *Pitta* vs. *Kapha* 1.000

* GFR—Glomerular filtration rate.

## Data Availability

This article includes the data generated and analyzed in this study.

## Supplementary Materials

The following supporting information can be downloaded at: https://www.mdpi.com/article/10.3390/diseases10010015/s1, Table S1 contains a brief overview of the *Prakriti* assessment tool; Table S2: Comparison of *Kapha Prakriti* between individuals with diabetes and without diabetes; Table S3: Comparison of *Pitta Prakriti* between individuals with diabetes and without diabetes.

## Limitation

The study covers a population of eastern Uttar Pradesh, India only. In the future, to obtain more precise results, the study may be conducted with a large sample size, and samples may be collected from a different area of the country or worldwide.
