# Supplementary material for: Clinical Prediction of Type 2 Diabetes Mellitus (T2DM) via Anthropometric and Biochemical Variations in Prakriti
Source: Diseases. 2022 Mar 3;10(1):15. doi: 10.3390/diseases10010015 (PMC8947277; doi:10.3390/diseases10010015)
Supplement: Supplementary file 1 [file diseases-10-00015-s001.zip › diseases-1591464-supplementary.pdf]

Clinical prediction of type 2 diabetes mellitus (T2DM) via anthropometric and biochemical variations in *Prakriti*

Shriti Singh[1*], Neeraj Kumar Agrawal [2], Girish Singh[3], Sangeeta Gehlot[1], Santosh Kumar Singh[4], Rajesh Singh[4*]

Affiliations:

[1] Department of Kriya Sharir, Faculty of Ayurveda, Institute of Medical Sciences, Banaras Hindu University, Varanasi 221005, Uttar Pradesh, India; sangeetagehlot@gmail.com
[2]Department of Endocrinology and Metabolism, Institute of Medical Sciences, Banaras Hindu University, Varanasi 221005, Uttar Pradesh, India; drnkavns@gmail.com
[3]Centre of Biostatistics, Institute of Medical Sciences, Banaras Hindu University, Varanasi 221005, Uttar Pradesh, India; drgirishsingh@yahoo.com
[4]Department of Microbiology, Biochemistry, and Immunology, Cancer Health Equity Institute, Morehouse School of Medicine, 720 Westview Drive SW, Atlanta, GA 30310, USA; sksingh@msm.edu

*Correspondence: singhshriti@gmail.com (S.S.); rsingh@msm.edu (R.S.); Tel: +1-404-756-6661 (R.S.); Fax: +1-404-752-1179 (R.S.)

Table S1: Overview of *Prakriti* assessment tool

PRAKRITI PARIKSHA

Vata	*Pitta*	*Kapha*
1. Your body is lean and thin<br>2. In your body, veins are prominent		1. You have a well-built body<br>2. You have long arms<br>3. You have a well-developed chest<br>4. Your forehead is broad
3. The colour of your skin is black or blackish red<br>4. Your skin is dry<br>5. Your skin has a tendency to be cracked.	1- The colour of your skin is fair or coppery<br>2. Your body temp generally remains in the higher range of normal<br>3. Your skin has moles and freckles	5. The colour of your skin is fair<br>6. Body temp in the lower range of normal<br>7. Your skin is smooth
6. Your hair is dry<br>7. Your hair has a tendency to be Cracked<br>8. Your hair is scarcely placed	4. Your hair is soft<br>5. Your hair has a yellowish tinge<br>6. Your hair is scarcely placed<br>7. You are suffering from premature greying of hairs<br>8. You are suffering from early hair fall	8. Your hair is black<br>9. Your hair is strong<br>10. Your hair is soft<br>11. Your hair is curly
9. Your nails are not smooth<br>10. Your nails are easily broken	9. Your nails are copper coloured	12. Your nails are whitish
11. Your extremities (palm and sole) have a tendency to be cracked	10. Your extremities are not compact	13. Your extremities are well developed

12. Your joints are not well covered with muscles<br><br>13. Your joints produce sound during movement	11. Your joints are loose	14. Your joints are well developed and covered with muscles
14. Your eyes are small<br><br>15. Your eyes are dusky<br><br>16. Your eyes remain open during sleep	12. Your eyes become red when exposed to the sun	15. Your eyes are whitish<br><br>16. You have long eyelashes
17. When you move with your friends, they lag behind you.		17. When you move with your friends, you lag behind them.
18. Does your voice is considered unpleasant.	13. Do you feel difficulty in pronunciation.	18. Does your voice is considered pleasant.
19. You are more talkative among your friends<br><br>20. When you have been asked some questions, you start with something else and come to the point instead of directly answering.	14. During the discussion, if individuals disagree with you, they become angry.	19. During the discussion, you speak on any topic<br><br>1. After long thinking<br><br>2. Avoid harsh words.
21. Sometimes you feel hungry and sometimes not<br><br>22. Sometimes you feel that meal is digested properly and sometimes not.	15. You always feel that you are very much hungry at mealtime<br><br>16. Your food is always digested properly.	20. You do not feel much hungry at mealtime 21. Your food is not properly digested.
23. You consume food materials in small quantity<br><br>24. Your frequency of food intake is more	17. You are not able to tolerate hunger<br><br>18. You consume a large quantity of food	22. You can tolerate hunger.
-	19. You feel increased thirst and consume an excessive quantity of water.	23. You feel thirsty not so often.

-	20. You suffer from excess sweat.<br>21. Your sweat is foul-smelling	-
25. You are frequently constipated and pass hard and dried stool	22. You pass a large quantity of stool<br>23. You pass a large quantity of Sweat	24. You pass less quantity of stool<br>25. You pass less quantity of sweat
	24. You pass an excessive quantity of urine	26. You pass less quantity of urine
26. You desire to eat hot food	25. You desire to eat cold food	27. You desire to eat hot food
27. You do not tolerate the winter season.	26. You do not tolerate the summer season.	28. You do not tolerate the winter season.
28. You like to eat food having<br>1-sweet<br>2-sour<br>3- salty taste	27. You like to eat food having<br>1-sweet<br>2- bitter<br>3-astringent taste.	29. You like to eat food having<br>1-spicy<br>2- bitter<br>3 -astringent taste.
29. You feel exhausted after light exertion.	28. You feel exhausted after moderate exertion.	30. You do not feel exhausted even after heavy exertion.
30. You frequently fall ill.	29. Do you suffer more from an abscess, moles, freckles, etc.	31. You do not frequently fall ill.
31. You have difficulty in falling asleep<br>32. Your sleep is disturbed many times		32. You tend to have a deep sleep.<br>33. You tend to sleep for long hours.

33. Whether you have a tendency to initiate the assigned work readily.		34. Whether you have a tendency to postpone the assigned work.
34. Do you have a tendency to collect others' belongings.<br>35. You are fearful in nature.<br>36. You tend to feel jealous about others' success.	30. Do you get easily angry.<br>31. Do you feel confident most of the time.	35. Do you not get angry easily?
37. Your grasping power is good.	32. You are considered intelligent.	36. Your retaining capacity is better than grasping power.
38. You tend to forget things easily.		37. Your retention power is good.
39. You tend to become anxious while under stress.	33. You are not able to tolerate even mild stress.	38. You can tolerate even severe stress.
40. You do not feel gratitude towards those who help you.	34. You tend to show mercy even on your enemies too.	39. You feel highly grateful towards those who help you.
41. You have no faith in God.	35. You have faith in God	40. You have a strong faith in God.
42. You avoid participating in competitive events	36. You enthusiastically participate in all competitive events	41. You enthusiastically participate in all competitive events
43. You frequently dream of flying, climbing mountains, trees, buildings, etc., during sleep.	37. You frequently dream of fire, electric thundering, red forest, etc., during sleep.	42. You frequently dream of ponds, lakes full of lotus and swan, clouds, rivers, and romantic scenes.
**Total Score.** ***Vata-***	***Pitta-***	***Kapha-***

Table S2: Comparison of *Kapha Prakriti* between individuals with diabetes and without diabetes

**Parameters**	***Kapha Prakriti* individuals with diabetes (n=60) (mean±SD)**	***Kapha Prakriti* individuals without diabetes (n=40) (mean±SD)**	**t-test**	**P-value**
Height(cm)	160.15 ± 10.17	165.30 ± 6.52	2.833	**0.006**
Weight(kg)	71.00 ± 10.33	67.57±10.05	1.644	0.103
BMI(kg/m^2)	27.68 ± 3.72	24.73 ± 3.23	4.086	**<0.001**
Systolic Blood Pressure (mm of Hg)	123.58 ± 6.03	114.98 ± 3.60	8.104	**<0.001**
Diastolic Blood Pressure (mm of Hg)	79.43±8.69	76.45±3.75	2.044	**0.044**
Waist Hip Ratio(cm)	0.96±0.054	0.95±0.09	0.557	0.579
Fasting Blood Sugar(mg/dl)	182.90 ± 77.70	97.37 ± 7.96	6.925	**<0.001**
Post Prandial Blood Sugar(mg/dl)	289.63 ± 112.13	123.16 ± 14.72	9.320	**<0.001**
HDL(mg/dl)	43.56 ± 7.73	44.00 ± 4.24	0.325	0.746
LDL(mg/dl)	126.29±38.50	98.53±19.84	4.199	**<0.001**
Triglyceride(mg/dl)	164.09±69.29	124.55 ±14.21	3.554	**0.001**
Total Cholesterol(mg/dl)	188.91± 39.74	164.56 ±18.39	3.620	**<0.001**
Serum Creatinine (mg/dl)	0.99±0.32	0.80±0.08	3.727	**<0.001**
eGFR (mL/min/1.73m^2)	77.40±26.26	100.38±7.84	5.368	**<0.001**

Table S3. Comparison of *Pitta Prakriti* between individuals with diabetes and without diabetes

Parameters	*Pitta Prakriti* individuals with diabetes (n=51) (mean±SD)	*Pitta Prakriti* individuals without diabetes (n=55) (mean±SD)	t-test	P- value
Height(cm)	158.22±7.25	163.16±7.56	3.423	**0.001**
Weight(kg)	63.69±11.47	62.82±9.85	2.471	**0.015**
BMI (kg/m^2)	25.52±4.45	23.77±2.95	0.341	0.734
Systolic Blood Pressure (mm of Hg)	135.14±16.22	117.78±3.84	7.706	**<0.001**
Diastolic Blood Pressure (mm of Hg)	82.12±10.19	78.62±4.03	2.355	**0.020**
Waist Hip Ratio(cm)	0.88±0.046	0.95±.078	5.053	**<0.001**
Fasting Blood Sugar(mg/dl)	142.48±30.97	90.78±8.17	11.945	**<0.001**
Post Prandial Blood Sugar(mg/dl)	219.85±63.88	116.35±9.85	11.870	**<0.001**
HDL (mg/dl)	39.09±7.25	41.16±4.06	1.835	0.069
LDL (mg/dl)	106.55±43.00	97.78±18.50	1.381	0.170
Triglyceride(mg/dl)	137.05±77.05	122.17±11.97	1.415	0.160
Total Cholesterol (mg/dl)	166.89±44.76	160.84±17.40	0.930	0.355
Serum Creatinine (mg/dl)	0.95±0.30	0.79±0.08	3.766	**<0.001**
eGFR (mL/min/1.73m^2)	83.02±40.32	99.23±7.06	2.936	**0.004**
